# The *M.3.2* mutation in *Drosophila melanogaster* mapped as a novel allele of ​​ *tout-velu*

**DOI:** 10.17912/micropub.biology.001489

**Published:** 2025-02-11

**Authors:** MacKenzie Patterson, Adriana R. Andrus, Conner Bell, Justin J. Bromell, Daniel Buchanan, Diana H. Chammout, Wisler X. Charles, Joseph E. Cummings, Anabelle Dabesh, Nixon Deleon Garcia, Kevin W. Dickerson, Toritsemose R. Emiko, Sara Fathi, Tavis J. Floyd, Hailee Gragg, Senai B. Griffin, Lauren Heininger, Amber Ibrahim, Cameron Ivey, Ahmad Jackson, Lamar Jackson, Deepthy Jeeva, Leland E. Jones, Kinaan Kana, Anthony S. Kemp, Ayyoob Khan, Megan Rose Kowal, Shane J. Kowaleski, Tristan C. Lago, Eli Lynch, Shirin Maalhagh Fard, Khalil Makki, Tanya Moaikel, Jordan M. Mosby, Dallas A. Ricketts, Reese J. Saho, Florine Sanchez, Caden H. Sauerbrey, Paul C. Sprowl, Kaitlyn M. Sullivan, Michael Thomas, Jaden J. Tripette, Chimezie Ugbaja, Gregory A. Volcy, Jamarr Watson, De'Andre Williams, Kayla L. Bieser, Alexandra Peister, Jamie Siders, Jacob D. Kagey

**Affiliations:** 1 University of Detroit Mercy, Detroit, Michigan; 2 Ohio Northern University, Ada, Ohio; 3 Morehouse College, Atlanta, Georgia; 4 Nevada State University, Henderson, Nevada, United States

## Abstract

In *Drosophila melanogaster* genetic screens are often used to identify genes associated with different biological processes. Here, we have utilized the Flp/FRT system to generate mitotic clones within the developing eye. These clones were screened for mutations that disrupt cell division, organ patterning, and cell growth. One such mutation from this screen, mutant M.3.2, resulted in an expansion of the cuticle within the area normally covered by ommatidium as well as an overall smaller eye size. Genetic and molecular mapping revealed this mutation to be in the gene, t *out-velu* ( *ttv* ).

**Figure 1. M.3.2 disrupts mosaic eye development and maps to the tout-velu locus.**
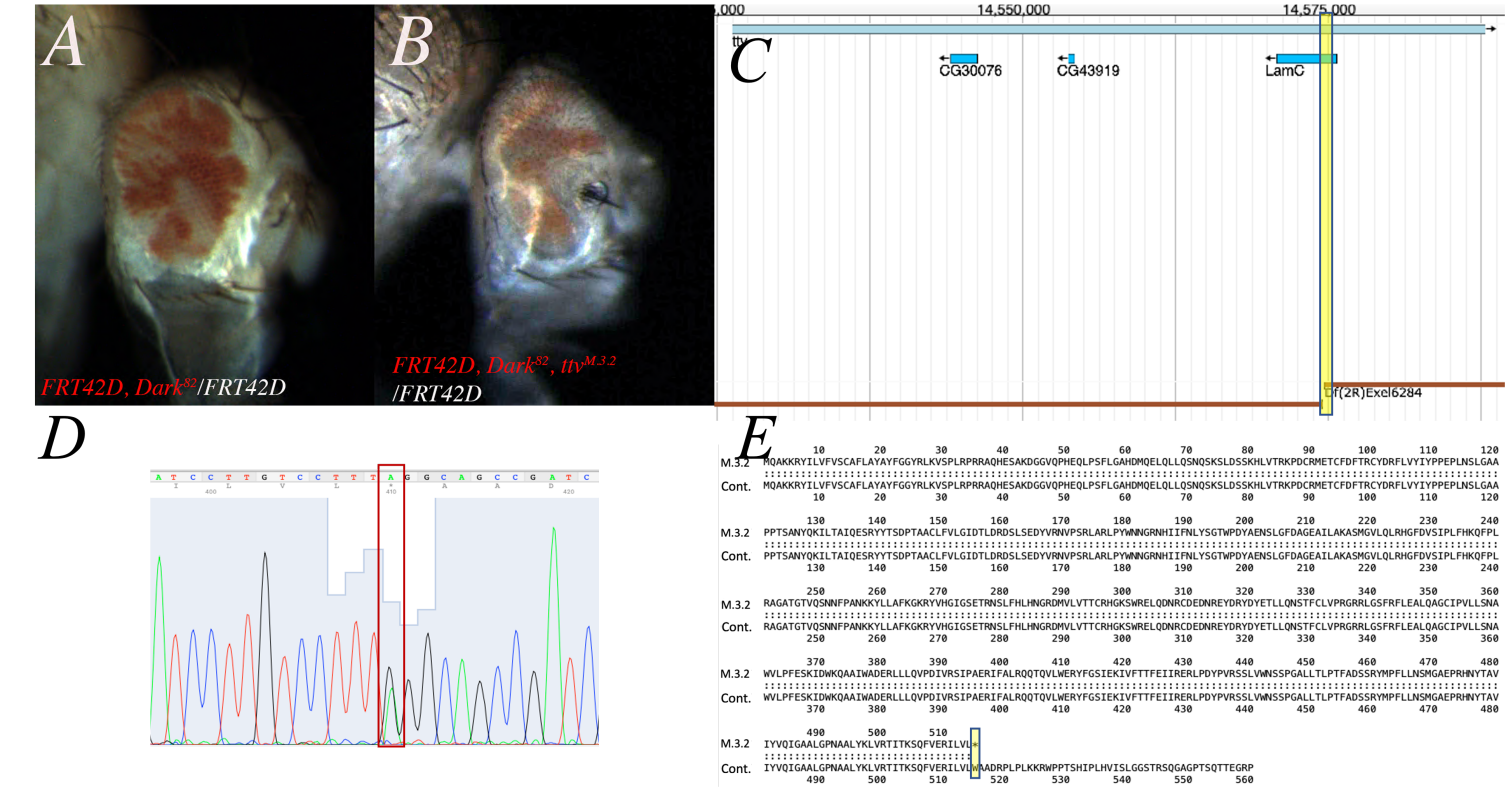
*M.3.2* is a truncation allele of *tout-velu* ( *ttv* ). A. Mosaic eye phenotype of *Dark ^82^* mosaic, pigmented tissue ( *mini-white ^+^* ) is mutant. B. Mosaic eye of *Dark ^82^ , ttv ^M.3.2^* , mutant tissue is pigmented ( *mini-white ^+^* ). Expanded cuticle and hair can be seen in double mutant mosaic eye. C. Genetic mapping of *M.3.2* results in two non-overlapping deficiencies that failed to complement leaving a region of 2R:14,574,557 and 14,574,750 that both fail to complement. Two genes span the gap between the two deficiencies. Image adapted from ​​FlyBase. D. Sanger sequencing of *Dark ^82^ , ttv ^M.3.2^* in *ttv* region reveals SNP at 2R:14583318 in *ttv* exon. E. M.3.2 mutations result in a truncation mutation of TTV. *M.3.2* sequence identified a nonsense mutation that shift from W to *. Image adapted from fasta.virginia.edu.

## Description

*Drosophila melanogaster* is a well-established genetic model system that has been an excellent source for gene discovery, in particular utilizing developing tissue(s) such as the eye (Morgan 1911) . All manners of genetic screening have been conducted in *Drosophila,* ranging from developmental screens to behavioral screens (St Johnston 2002) . A Flp/FRT EMS screen was conducted to identify developmental cell growth and cell division regulators on chromosome 2R. This screen identified mutations that disrupt cell growth, cell cycle, and organ development (Kagey *et al.* 2012). Additionally, the screen was conducted in a genetic background in which cell death is homozygous blocked (via the *Dark ^82^* allele present on the starting chromosome) (Akdemir *et al.* 2006). Here, students from the Fly-CURE consortium worked within undergraduate laboratory courses to characterize and map one mutant from this screen, mutant *M.3.2* (Merkle *et al.* 2023).

Mosaic eyes were generated by mating the control (starting chromosome) *;FRT42D, Dark ^82^ /CyO* and *;FRT42D, Dark ^82^ , M.3.2/CyO* to virgin females of *;FRT42D, Ey-Flp* (BDSC). The *Dark ^82^* allele was generated with a *mw+* containing transposon, so the *Dark ^82^* containing mutant tissue is pigmented in both eyes. Straight-winged F1 progeny were scored for their mosaic eye phenotype. As previously reported, the *Dark ^82^* mosaic eye results in a slight over representation of red tissue to white tissue (60-70% pigmented tissue, and Figure 1A ) (Akdemir *et al.* 2006; Kagey *et al.* 2012). Contrastingly, the *Dark ^82^ , M.3.2* mosaic eye results in a deformed mosaic eye that has an expansion of cuticle, additional hair in the eye, and an overall smaller eye size than the control ( Figure 1A compared with 1B). The expansion of the cuticle coupled with the overall smaller adult eye size, suggests that there may be a non-autonomous aspect of the *M.3.2* mutation.

The genetic mapping of the location of mutant *M.3.2* was done by three independent groups of researchers within undergraduate genetics laboratory courses at Ohio Northern University, Morehouse College, and the University of Detroit Mercy as part of the Fly-CURE consortium (Merkle *et al.* 2023). Genetic mapping was accomplished by setting up complementation tests between the *Dark ^82^ , M.3.2* mutant stocks and the 2R deficiency kit (BDSC) (Cook *et al.* 2012). Virgin females from the *;FRT42D, Dark ^82,^ M.3.2 / CyO* stock were mated in series to male flies from the 87 deficiency stocks that comprise the Bloomington Stock Center 2R Deficiency kit (and are distal to the FRT42D location). Triplicate data from the three groups of mapping students confirmed that M.3.2 failed to complement *Df(2R) Exel6284* and *Df (2R) ED2354.* Although these two deficiencies do not overlap ( overlap Chr2R:14,574,557 and14,574,750) there are two genes that span this gap, leaving a region that fails to complement of only two predicted genes ( Figure 1B, yellow box, and Table 1).

Further genetic mapping found that the M.3.2 mutation failed to complement two independent alleles of the gene *tout-velu (ttv ^00681b^ and ttv ^k11904^ )* , suggesting that *M.3.2* is a novel allele of *tout-velu, ttv ^M.3.2^* . Once we had identified the causative locus for the *M.3.2* mutation, we utilized WGS of heterozygous flies to look for the specific heterozygous mutations within the *ttv* locus that are unique to M.3.2 and not present in any other 2R mutant we have sequenced Bieser et al. (in preparation). From this we identified a point mutation in the *ttv* locus that introduces a stop codon at the 515 amino acid (Trp515*) ( Figure 1C ). TTV is a 772 amino acid protein and having a truncation at amino acid 515 suggests that the *M.3.2* mutation in *ttv* is a truncation mutation and a novel null allele of *ttv* ( Figure 1C ). Given the extensive genetic and molecular data presented here we can reasonably conclude that *M.3.2* is a lethal (and novel) allele of *ttv* and there are likely no additional lethal mutations (other than *Dark ^82^* ) on the chromosome. However, a future rescue experiment would be necessary to demonstrate the phenotype observed is based solely on the *ttv* and *Dark* mutations.

*Tout-velu* is a homologue of the human gene, *EXT1* , which is a tumor suppressor needed for the Hedgehog protein diffusion (Kim *et al.* 2001). In flies, Hedgehog signaling is essential for proper development of many different tissues, including the processing of the morphogenetic furrow in the developing eye (Heberlein *et al.* 1993). Given that previous studies have found a non-autonomous role for TTV in promoting Hh signaling, and the non-autonomous mosaic phenotypes we observed, we provide additional data supporting a non-autonomous role of TTV function (Bellaiche *et al.* 1998). The Hedgehog protein is a morphogen essential for embryonic development, cell growth, cell specialization, and the patterning or shaping of the body, as TTV ensures receiving cells can interpret Hh signaling. Thus, it is likely that the *ttv ^M.3.2^* mutant clones disrupted the MF progression in the developing eye both autonomously and non-autonomously. Additionally, loss of TTV results in cells that are unable to interpret Hh signaling from the MF, resulting in loss of Hh signaling for homozygous mutant cells, which would lead to arrested development of ommatidia (Ma and Moses 1995) .

Lastly, the inclusion of the *Dark ^82^* allele allowed for the discovery of this *ttv* allele as a conditional regulator of cell growth and cell division in this Flp/FRT system. Previous studies disrupting Hh signaling in the developing eye through mutations in *Ptc* , *Cos2* , or *Smo* have resulted in both autonomous and non-autonomous apoptosis (Christiansen *et al.* 2012; Kagey *et al.* 2012; Moore *et al.* 2022). Given that *ttv* mutations disrupt Hh signaling, it is reasonable to suggest that *ttv ^-/-^* clones would undergo apoptosis, and thus may have been missed in the initial round of Flp/FRT growth mutants (Moberg *et al.* 2001; Tapon *et al.* 2002; Harvey *et al.* 2003). This would define *ttv ^M.3.2^* as a conditional negative regulator of cell growth and development, only identified in a genetic background of blocked apoptosis (Gilbert *et al.* 2011; Kagey *et al.* 2012).

## Reagents

**Table:** 

Starting stock for 2R EMS screen			
**Gene**	**BDSC #**	**Genotype**	**Citation**
*Dark*	23285	FRT42D,Dark ^82^ /CyO	Akdemir *et al.* 2006
Stocks from BDSC 2R Deficiency Kit			
**Genotype**	**BDSC #**	**Region**	**Complementation Results**
*Df(2R)ED2354*	8913	2R:14259045..14574557	Failure to complement
*Df(2R)Exel6284*	7749	2R:1457750..14765770	Failure to complement
Additional BDSC Deficiency stocks tests (not in 2R kit)			
*Df(2R)BSC357*	24381	2R:14363886..14713687	Failure to complement
*Df(2R)Exel8059*	7877	2R:14458207..14575583	Failure to complement
Single genes tested for complementation			
**Gene**	**BDSC**	**Allele**	**Complementation Results**
*ttv*	10949	*ttv ^00681b^*	Failure to complement
*ttv*	11050	*ttv ^k11904^*	Failure to complement
